# From gene to heart: the impact of a novel *SGCD* variant in familial dilated cardiomyopathy

**DOI:** 10.1186/s12920-026-02342-5

**Published:** 2026-03-17

**Authors:** Samira Kalayinia, Amirhossein Poopak, Mahdieh Soveizi, Majid Maleki

**Affiliations:** 1 Cardiogenetic Research Center, Rajaie Cardiovascular Institute, Tehran, Iran; 2 Cardiovascular Research Center, Rajaie Cardiovascular Institute, Tehran, Iran

**Keywords:** Dilated Cardiomyopathy, SGCD, Whole Exome Sequencing, δ-Sarcoglycan Complex, Autosomal Dominant Inheritance

## Abstract

**Background:**

Dilated cardiomyopathy (DCM) is a leading cause of heart failure, often resulting in reduced ejection fraction and progressive cardiac dysfunction. Although up to half of idiopathic DCM can be linked to genetic variants, many familial cases still lack a definitive molecular diagnosis. Sarcoglycan delta (*SGCD*) encodes a crucial component of the dystrophin-glycoprotein complex, and variants in this gene have been implicated in both muscular dystrophies and cardiomyopathies.

**Methods:**

We evaluated a three-year-old girl presenting with a confirmed diagnosis of DCM. Clinical assessments included echocardiography and cardiac magnetic resonance imaging (CMR), revealing moderate-to-severe systolic and diastolic dysfunction. Whole exome sequencing (WES) was performed to investigate potential causative variants. In silico analysis and Sanger sequencing were used to confirm and characterize any identified alteration in the proband and her parents.

**Results:**

WES identified a novel heterozygous *SGCD* variant, NM_000337.6(SGCD): c.647 A > T, p.(Asn216Ile), located in exon 8. Sanger sequencing confirmed this variant’s presence in the proband and her father, suggesting a familial inheritance pattern. In silico predictive tools supported a likely deleterious effect of the variant,, with functional analysis indicating possible disruption of δ-Sarcoglycan’s structure. This variant was absent in public variant databases, underscoring its rarity. Comparative evaluation of known *SGCD* variants further highlighted exon 8 as a possible mutational hotspot in DCM.

**Conclusion:**

These findings expand the variant spectrum of *SGCD* and reinforce its role in familial DCM. Genetic screening for *SGCD* variants in individuals with idiopathic or familial cardiomyopathy can improve early diagnosis, guide targeted interventions, and inform genetic counseling. Our results underscore the clinical importance of integrating molecular diagnostics to enhance personalized management of DCM.

## Introduction

Dilated cardiomyopathy (DCM) is one of the most critical causes of heart failure worldwide, characterized by dilation and impaired contraction of the left or both ventricles [1]. DCM has a heterogeneous nature and diverse clinical presentations, which often make it challenging to diagnose and treat [2]. In many cases, patients remain asymptomatic for years before developing overt heart failure symptoms, which can rapidly progress and lead to significant morbidity and mortality [3]. Given this clinical complexity, a deeper understanding of the underlying genetic determinants is crucial for improving diagnostic accuracy and therapeutic approaches. Familial DCM accounts for approximately 20–30% of all DCM cases, has garnered increasing attention due to advancements in genetic testing [3, 4]. Today, up to half of idiopathic DCM cases can be identified through extensive genetic analysis, underscoring the importance of exploring hereditary causes. More than 50 genes have been implicated in DCM, yet the exact molecular culprits remain elusive for many families [5, 6]. This knowledge gap points to the necessity of uncovering novel variants and interpreting their functional consequences to inform genetic counseling and precision medicine better. Familial DCM accounts for about 20–30% of all DCM cases and is most commonly inherited in an autosomal dominant, age‑dependent, and often incompletely penetrant manner. Pathogenic variants are enriched in genes such as *TTN*, *LMNA*, *MYH7*, *DSP*, *FLNC* and *RBM20*, which together explain a substantial proportion of genetically confirmed familial DCM. Clinically, familial DCM presents with progressive heart‑failure symptoms and arrhythmic risk, and management combines guideline‑directed heart‑failure therapy with appropriate device implantation and systematic family screening [7]. The *SGCD* gene is located on chromosome 5q33–q34 and encodes δ-sarcoglycan, a transmembrane component of the sarcoglycan subcomplex within the dystrophin–glycoprotein complex that links the cytoskeleton to the extracellular matrix in striated muscle. SGCD transcripts are predominantly expressed in skeletal and cardiac muscle, and δ-sarcoglycan interacts with other sarcoglycans and dystrophin to maintain sarcolemmal stability. Biallelic loss-of-function *SGCD* variants cause autosomal recessive limb-girdle muscular dystrophy type 2 F, frequently accompanied by dilated cardiomyopathy, whereas heterozygous missense variants in the extracellular region have been associated with isolated or predominant cardiomyopathy, sometimes with arrhythmic phenotypes, supporting a genotype–phenotype spectrum that includes both muscular dystrophy and primary DCM [8]. In this context, viral infections such as COVID-19 are increasingly recognized as potential triggers that can unmask or aggravate pre-existing, genetically determined susceptibility to DCM. While left ventricular dysfunction has been reported during and after COVID-19 hospitalization in otherwise healthy individuals, distinguishing purely infection-related myocardial injury from the manifestation of an underlying familial cardiomyopathy can be challenging in clinical practice [9]. In this study, we report a novel heterozygous *SGCD* variant in a family that may be strongly associated with DCM.

## Materials and methods

### Study design and participants

**Fig. 1:**
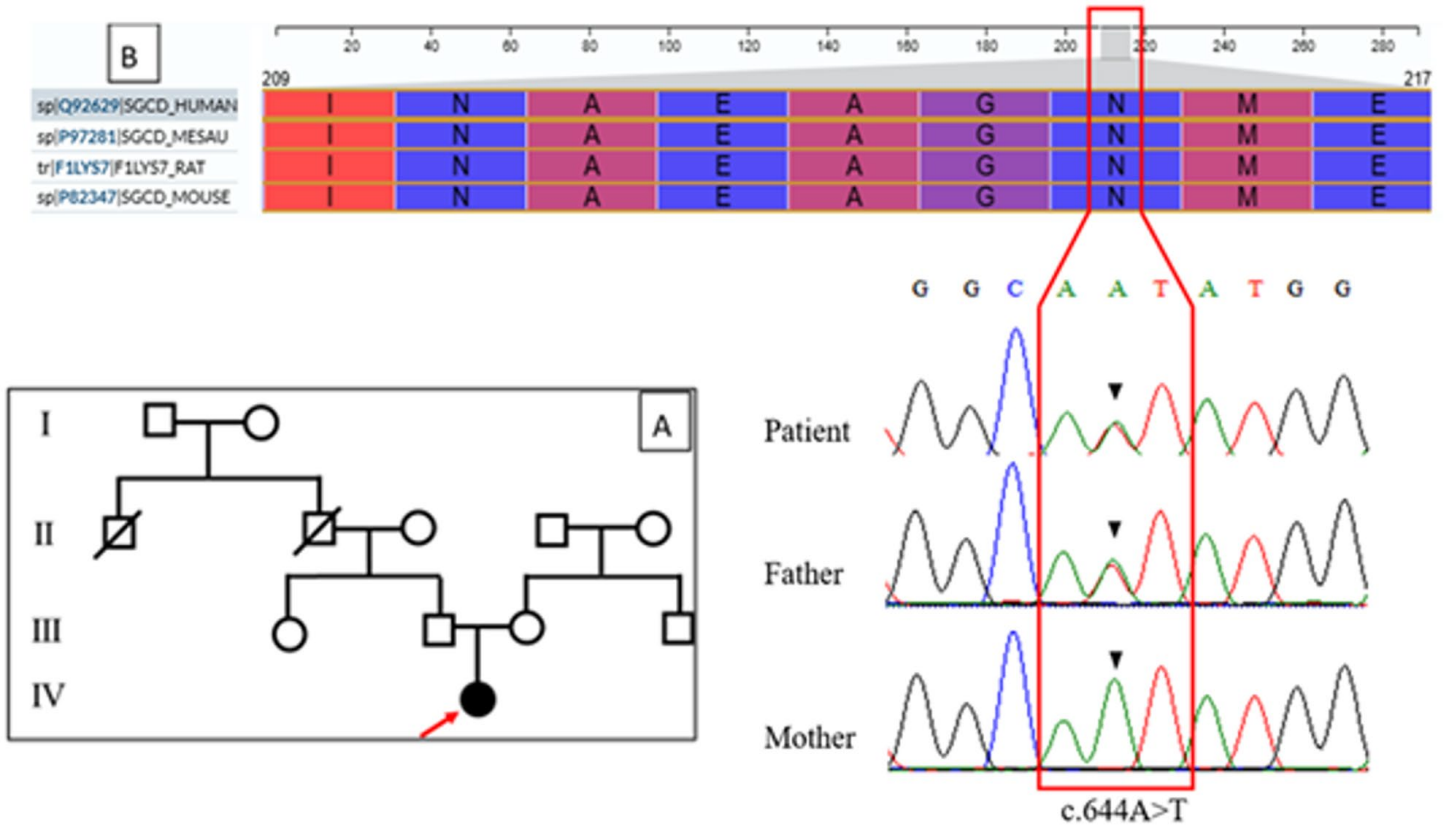
Family pedigree and conservation of the affected SGCD residue. **A** Pedigree of the family showing the proband (III-1, filled symbol) with early-onset dilated cardiomyopathy and her relatives. The heterozygous *SGCD* variant is present in the proband and her father (II-1), consistent with autosomal dominant transmission, while the mother (II-2) carries the wild-type allele. **B** Multi-species sequence alignment of δ-sarcoglycan highlighting the affected residue (Asn215/Asn216, indicated by an arrow), which is highly conserved across vertebrate species. The strong evolutionary conservation of this position supports its potential functional importance in the SGCD protein

A 3-year-old girl from non-consanguine parents, with a history of nausea and vomiting was admitted to a hospital (Fig. 1A). The patient’s COVID PCR tested positive, and during her hospitalization course, she experienced ejection fraction (EF) reduction and welfare worsening. The medical team initially diagnosed multisystem inflammatory syndrome in children (MIS-C) and post-COVID myocarditis. However, further echocardiographic and cardiac magnetic resonance imaging (CMR) evaluations, were conducted to ensure the accuracy of the diagnosis. Echocardiography evaluations presented moderate to severe global myocardial systolic and diastolic dysfunctions (EF: 30–35%) with a mild left ventricular (LV) hyper trabeculation. Based on these findings, the patient was ultimately diagnosed with DCM. At this time, the patient was referred to the Rajaie Cardiovascular Institute, Tehran, Iran, in 2023 for genetic counseling and further evaluation. Blood samples were taken from the patient and her family for genetic assessment Our study adhered to the Declaration of Helsinki and was approved by the Ethics Committee of Rajaie Cardiovascular Institute, Tehran, Iran (IR.RHC.REC.1404.087). Informed consent to participate was obtained from the parents or legal guardians of any participant under the age of 16 and clearly state this in your manuscript.

### Genetic analysis

Genetic counseling was conducted for the patient referred to the Cardiogenetic Research Center, Rajaie Cardiovascular Institute, who had been clinically diagnosed with DCM. Informed consent was obtained for sampling and subsequent genetic testing. Whole blood samples were collected, and DNA was extracted using the salt precipitation method [10]. The purity and concentration of the DNA sample were assessed with a NanoDrop spectrophotometer. The patients’ DNA was then prepared for whole exome sequencing (WES), with analysis focused on genes known to be involved in cardiomyopathies, using Illumina HiSeq 6000 platform (Illumina, San Diego, CA, USA) at a mean read depth of 200×. The raw data (FASTQ files) were processed for variant analysis. Variant calling and annotation were performed using a validated clinical exome pipeline. Reads were aligned to the human reference genome (GRCh37/hg19) and variants were called using validated in‑house procedures, followed by annotation with population, functional and disease databases. We applied quality filters requiring a minimum depth of coverage (e.g. ≥20×) and high genotype quality for all reported variants, and restricted further analysis to rare protein‑altering variants (missense, nonsense, frameshift, canonical splice‑site) with a minor allele frequency < 1% in gnomAD v4 and 1000 Genomes [11, 12]. Candidate variants were then prioritized within a curated cardiomyopathy/arrhythmia gene panel (including *TTN*, *LMNA*, *MYH7*, *TNNT2*, *DSP*, *FLNC*, *RBM20*, *PLN*, *SCN5A* and others) based on predicted deleteriousness, gene–disease validity, and consistency with the patient’s phenotype, and were classified according to ACMG/AMP guidelines. To confirm the accuracy of the test results, the identified variant was further examined in patient and her family members using Sanger sequencing. For this purpose, specific primers targeting the region of interest were designed with GeneRunner software (v.6.5.52 Beta) and verified using the Primer-BLAST tool (https://www.ncbi.nlm.nih.gov/tools/primer-blast). Subsequently, PCR amplification was performed, and then sequenced via ABI Sequencer 3500XL PE (Applied Biosystems, USA). Chromatograms were analyzed using BioEdit software (v.7.0.5.3).

### In silico studies

The identified variant’s frequency was assessed using the 1000 Genomes Project (http://www.1000genomes.org) and the Genome Aggregation Database (gnomAD) database (https://gnomad.broadinstitute.org/news/2023-11-gnomad-v4-0/). Subsequently, the pathogenicity of the variant was evaluated utilizing multiple web-based tools, including MutationTaster (https://www.mutationtaster.org), and CADD (https://cadd.gs.washington.edu). Furthermore, the alternation impact at the amino acid level and signaling pathway were investigated using the Enrichr (https://maayanlab.cloud/Enrichr), KEGG (https://www.genome.jp/kegg/pathway.html) and HOPE (https://www3.cmbi.umcn.nl/hope). The domain structure and the conservation of the wild-type amino acid across different species were visualized by UniProt (https://www.uniprot.org). For pathway enrichment analysis (Table 1), the *SGCD* gene was used as input in Enrichr, querying the KEGG “Dilated cardiomyopathy” pathway to assess its involvement in relevant signaling networks. The KEGG database was accessed in January 2025.

**Table 1:** Pathway enrichment analysis of *SGCD* and related genes using KEGG

Index	Name	*P*-Value	Adjusted *P*-Value	Gene Pathway
1	Viral myocarditis	1.81E-19	6.68E-18	*SGCG*,* SGCA*,* SGCD*,* SGCB*,* DMD*,* ACTG1*,* TNC*,* MYH6*,* MYH7*,* LAMINC*
2	Hypertrophic cardiomyopathy	5.46E-18	1.01E-16	*SGCG*,* SGCA*,* SGCD*,* SGCB*,* DMD*,* ACTG1*,* TNC*,* MYH6*,* MYH7*,* LAMINC*
3	Dilated cardiomyopathy	9.33E-18	1.15E-16	*SGCG*,* SGCA*,* SGCD*,* SGCB*,* DMD*,* ACTG1*,* TNC*,* MYH6*,* MYH7*,* LAMINC*
4	Arrhythmogenic right ventricular cardiomyopathy	5.54E-13	5.12E-12	*SGCG*,* SGCA*,* SGCD*,* SGCB*,* DMD*,* ACTG1*,* LAMINC*
5	Thyroid hormone signaling pathway	2.51E-05	0.000186	*ACTG1*,* MYH6*,* MYH7*
6	Cardiac muscle contraction	0.000823	0.005074	*MYH6*,* MYH7*
7	Adrenergic signaling in cardiomyocytes	0.002417	0.01278	*MYH6*,* MYH7*
8	cGMP-PKG signaling pathway	0.002985	0.0138	*MYH6*,* MYH7*
9	Focal adhesion	0.004289	0.01763	*TNC*
10	Vibrio cholerae infection	0.02473	0.09149	*ACTG1*

Each pathway is ranked by p-value and adjusted p-value, highlighting significant associations of the sarcoglycan family and other cardiac genes (e.g., *DMD*,* ACTG1*,* MYH6*) with dilated cardiomyopathy and related cardiovascular pathways

### Search strategy of *SGCD* variants

All reported variants in the *SGCD* gene associated with DCM and other types of cardiomyopathies were compiled using Human Gene Mutation Database (HGMD), along with additional information from the ClinVar, Franklin and The Leiden Open Variation Database (LOVD). These variants were subsequently evaluated for their pathogenicity and frequency across genomic databases. All databases and tools were accessed with the following versions: HGMD Professional (release 2024.1), ClinVar (accessed June 2025), LOVD v3.0, UniProt release 2025_02, 1000 Genomes (phase 3), gnomAD v4 (accessed Month Year), v4, CADD v1.6, and MutationTaster2021.

## Results

### Clinical phenotype

**Fig. 2:**
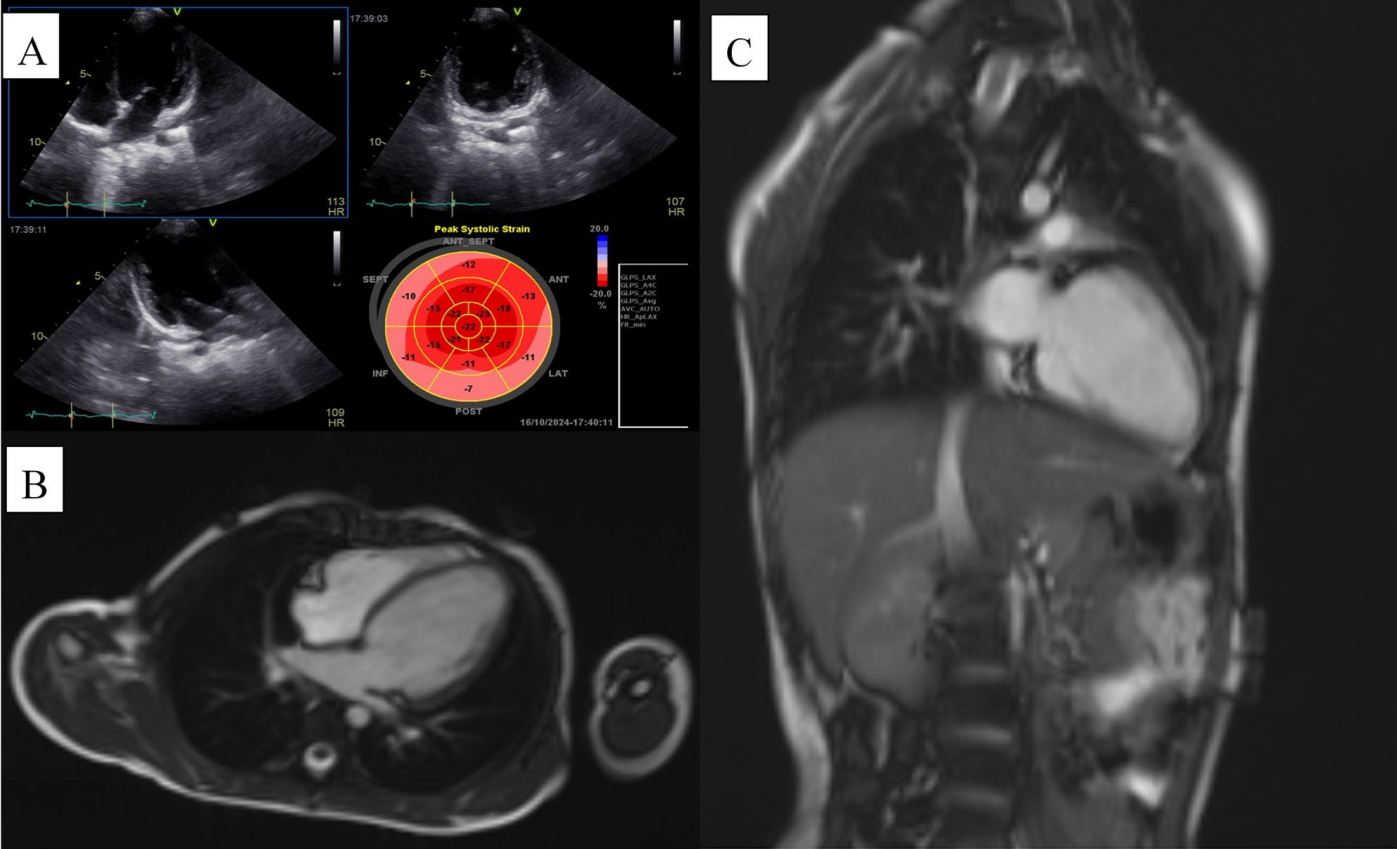
Cardiac imaging findings in the proband. **A** Transthoracic echocardiographic views demonstrating a dilated left ventricle with globally reduced systolic function; the bull’s-eye strain plot shows markedly decreased global longitudinal strain, consistent with severe impairment of myocardial contractility. **B** Axial cardiac magnetic resonance (CMR) image revealing left ventricular dilation with a thin myocardial wall and globally depressed systolic function. **C** Coronal CMR image further illustrating the enlarged, compromised left ventricle in keeping with a diagnosis of dilated cardiomyopathy. Together, these imaging findings confirm significant structural and functional remodeling of the left ventricle in the affected child

A three-year-old girl presented was admitted to the hospital. At admission, the child presented with persistent nausea and vomiting, poor oral intake, and progressive fatigue, but without prior history of congenital heart disease or known cardiac symptoms. Physical examination revealed tachycardia, mild tachypnea, hepatomegaly, and a soft systolic murmur, while peripheral perfusion was slightly reduced but there were no signs of overt peripheral edema. Baseline electrocardiography showed sinus tachycardia with non‑specific ST‑T changes but no sustained arrhythmias or conduction block. Laboratory tests were notable for elevated inflammatory markers and mildly increased cardiac biomarkers, consistent with a multisystem inflammatory response. During hospitalization, she received guideline‑directed heart‑failure therapy and immunomodulatory treatment for suspected MIS‑C/post‑COVID myocarditis, with partial improvement in symptoms but persistent left ventricular systolic dysfunction on follow‑up imaging, supporting the diagnosis of DCM. During hospitalization, she experienced a notable decline in ejection fraction (EF), accompanied by a progressive deterioration in overall clinical status. Initially, multisystem inflammatory syndrome in children (MIS-C) or post-COVID myocarditis was suspected. However, further cardiac assessments, including echocardiography and cardiac magnetic resonance imaging (CMR) revealed moderate to severe global systolic and diastolic dysfunction (EF: 30–35%) as well as mild left ventricular hypertrabeculation, thereby supporting a diagnosis of DCM Fig. 2. Although her initial symptoms were nonspecific (primarily nausea and vomiting), more advanced cardiac workup uncovered a structural and functional pathology consistent with DCM. At the time of referral to our institute, the proband had no overt signs of arrhythmic events, such as premature ventricular complexes or sustained tachyarrhythmias. However, her marked EF reduction and global functional decline signaled a progressive disease course requiring close monitoring. Given the suspicion of a hereditary cardiomyopathy, a genetic assessment was performed. Blood samples were obtained from the proband and her family members.

### Identification of Novel *SGCD* Variant

After filtering WES data against our cardiomyopathy/arrhythmia gene panel and population databases (gnomAD v4, 1000 Genomes), the *SGCD*, NM_000337.6(SGCD): c.647 A > T, p.(Asn216Ile), variant emerged as the only rare, protein‑altering variant meeting our pathogenicity criteria in the analyzed genes. A novel heterozygous alternation (c.644 A > T) in the *SGCD* gene was identified through the WES analysis Fig. 1A, B. This variant is located in exon 8 at the chromosomal position; chr5:156184663 A > T, resulting in an amino acid substitution from Asparagine (N) at the position 215 to Isoleucine (I) at the protein level p.(Asn216Ile). In-silico analysis indicated that this variant has not been previously reported in the Iranome database, gnomAD, or the 1000 Genomes projects. In addition, it has been classified as a disease-causing variant in MutationTaster software, and CADD analysis assigned it a Phred score of 24.3, suggesting potential pathogenicity. The results of Sanger sequencing in patient and her parents confirmed the identified variant by PCR with specific primers (Forward: AGAAGCAGGACAAAGTAGAAAGG, and Reverse CTGGGACTGTGTTGGCATA). Parental genotypic analysis revealed that the father was heterozygous for this alternation, while the mother carried the wild type allele, supporting paternal inheritance Fig. 1B. Based on ACMG/AMP classification, this variant is currently categorized as a variant of uncertain significance (VUS).

### Functional impact

**Fig. 3:**
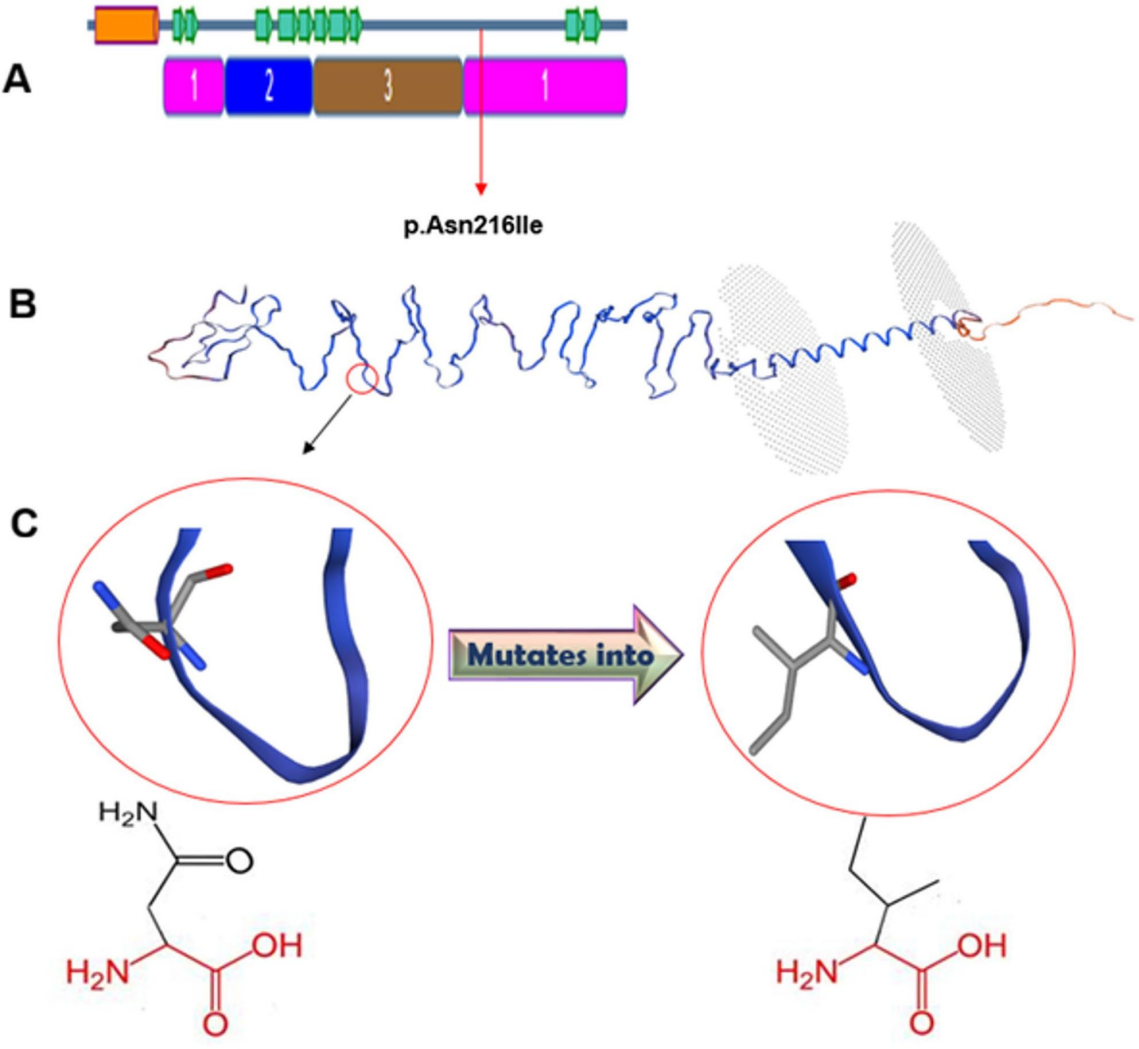
Predicted structural impact of the SGCD p.Asn216Ile variant. **A** HOPE output showing the wild-type residue (Asn) forming a turn within the extracellular region of δ-sarcoglycan. **B** Predicted structure with substitution to Ile, indicating loss of hydrogen-bonding capacity and increased local hydrophobicity, which may destabilize the conformation. **C** Summary of predicted physicochemical changes (size, charge, hydrophobicity) associated with the Asn→Ile substitution

The alignment of amino acid sequences derived from UniProt for Homo sapiens, compared to other species, indicated that Asparagine (N) at position 215 of the 289-amino-acid SGCD protein is highly conserved Fig. 1B. Additionally, the mutant residue is located near a highly conserved region, suggesting potential functional significance. Given that each amino acid has distinct biochemical properties—including size, charge, and hydrophobicity-value—this change might lead to disruptions in protein interactions, loss of hydrogen bonding, and/or misfolding of the protein structure Fig. 3. Furthermore, an analysis of the KEGG signaling database via Enricher for the *SGCD* gene revealed that *SGCD* plays a crucial role in DCM, with a highly significant p-value of 9.331e-18, as well as in other cardiomyopathies, reinforcing its potential pathogenic role in cardiac disease Table 1.

### Comparison with other variants

We compiled a comprehensive table summarizing all reported variants from several key databases, including the Human Gene Mutation Database (HGMD), ClinVar, and the Leiden Open Variation Database (LoVD). The distribution of variants across different exons of the *SGCD* gene was analyzed, revealing a notable concentration of reported variants within specific exons, particularly exons 4, 6, 8, and 9. This pattern suggests that these regions may be hotspots for variants. Our variant of interest is also located in exon 8. The presence of multiple variants in this exon highlights its potential role in the pathogenesis of associated disorders. In addition, among the 41 reported variants, 12 were associated with DCM Table 2.

**Table 2:** Summary of reported mutations in *SGCD* gene (NM_001128209)

No.	Mutation type	Gene Position	Exon	Nucleotide change	Protein change	dbSNP	Phenotype	Frequency				Pathogenesity			Ref
								1000G	ExAC	ClinVar	LoVD	MutationTaster	CADD	ACMG	
1	Missense	chr5-156344500 G > C	3	c.15G > C	p.Glu5Asp	rs549319429	SCD	0	0	B/LB	LB/VUS	DC	17.4	B	[13]
2	Nonsense	chr5-156344574 G > A	3	c.89G > A	p.Trp30*	rs121909296	LGMD	0	0	P	P	DC	46	P	[14]
3	Nonsense	chr5-156344582 C > T	3	c.97 C > T	p.Arg33*	rs778760498	LGMD2	0	0	P	NA	DC	37	P	[14]
4	Nonsense	chr5-156508610 G > T	4	c.202G > T	p.Gly68*	-	LGMD	0	0	NA	P	DC	43	LP	[15]
5	Missense	chr5-156508620 G > C	4	c.212G > C	p.Arg71Thr	rs776240936	DCM	0	0	NA	P	DC	28.7	VUS	[16]
6	Missense	chr5-156508634 G > T	4	c.226G > T	p.Gly76Cys	rs376659221	LGMD2F	0	0	B	P	DC	31	LP	[17]
7	Nonsense	chr5-156508685 G > T	4	c.277G > T	p.Glu93*	-	LGMD	0	0	NA	P	DC	48	LP	[18]
8	Missense	chr5-156508698 G > A	4	c.290G > A	p.Arg97Gln	rs45559835	DCM	0	0	B/LB	B/LB	B	24.4	B	[19]
9	Nonsense	chr5-156508697 C > T	4	c.289 C > T	p.Arg97*	rs758700138	LGMD2F	0	0	P	P	DC	39	P	[20]
10	Missense	chr5-156589234 A > C	5	c.298 A > C	p.Asn100His	rs1436273126	LVNC	0	0	VUS	NA	DC	23.2	VUS	[21]
11	Missense	chr5-156594940 G > C	6	c.391G > C	p.Ala131Pro	rs267607045	LGMD	0	0	P	P	DC	18.4	VUS	[22]
12	Missense	chr5-156594943 G > A	6	c.394G > A	p.Val132Ile	rs367819390	DCM	0	0	LB/VUS	VUS	DC	12.4	VUS	[23]
13	Missense	chr5-156595000 T > G	6	c.451T > G	p.Ser151Ala	rs121909298	DCM	0	0	VUS	LB/P/VUS	DC	23.5	VUS	[23]
14	Nonsense	chr5-156595042 C > T	6	c.493 C > T	p.Arg165*	rs121909295	LGMD	0	0	P	P	DC	35	P	[14]
15	Nonsense	chr5-156647529 G > T	7	c.568G > T	p.Glu190*	rs1412814368	LGMD	0	0	P	P	DC	48	P	[24]
16	Missense	chr5-156757598 G > C	8	c.593G > C	p.Arg198Pro	rs750459196	LGMD	0	0	VUS	P	DC	32	LP	[25]
17	Missense	chr5-156757615 G > T	8	c.610G > T	p.Ala204Ser	-	IVF	0	0	NA	NA	DC	27.9	VUS	[26]
18	Missense	chr5-156757636 A > T	8	c.631 A > T	p.Asn211Tyr	-	LGMD	0	0	NA	VUS	DC	23.7	VUS	[27]
19	Missense	chr5-156759248 C > T	9	c.731 C > T	p.Pro244Leu	rs375159661	DCM	0	0	VUS	VUS	DC	21.8	VUS	[23]
20	Missense	chr5-156759301 G > A	9	c.784G > A	p.Glu262Lys	rs121909297	LGMD	0	0	LP	P	DC	32	LP	[28]
21	Missense	chr5-156759356 C > A	9	c.839 C > A	p.Ser280Tyr	rs397516337	DCM	0	0	VUS	VUS	DC	26.9	VUS	[23]
22	Missense	chr5-156759365 A > G	9	c.848 A > G	p.Gln283Arg	rs397516338	DCM	0	0	VUS/LB	LB	DC	23.5	B	[29]
23	Splicing	chr5-156647462 A > G	IVS6	c.503–2 A > G	-	rs1296549667	LGMD	0	0	NA	NA	DC	32	LP	[30]
24	Intronic	chr5-156757722 C > G	IVS8	c.699 + 18 C > G	-	rs180898690	TAPVR	0	0	VUS/B/LB	LB	B	5.8	B	[31]
25	Deletion	chr5:155753657_155753670delTGTAATAAGCTTGT	5`UTR	c.−626_−616del	-	rs149976574	DCM	0	0	B/LB	NA	DC	1.6	B	[29]
26	Deletion	chr5-156344605 GCTC > G	3	c.124_126del	p.Leu42del	-	Myopathy	0	0	NA	NA	DC	20	VUS	[32]
27	Deletion	chr5-155935663 ACT > A	4	c.245_246delCT	p.Ser82*	rs1561743757	LGMD	0	0	NA	P	DC	33	P	[24]
28	Deletion	chr5-156589274 AC > A	5	c.336_336delC	p.Asn112Lysfs*10	-	Myopathy	0	0	NA	NA	DC	27.7	P	[33]
29	Deletion	chr5-156021945 CA > C	6	c.387_387delA	p.Ala129Profs*2	rs397517921	DCM	0	0	P/LP	NA	DC	27.9	P	[34]
30	Deletion	chr5-156021967 TA > T	6	c.411_411delA	p.Lys137Asnfs*4	rs1383430588	LGMD	0	0	NA	NA	DC	32	P	[30]
31	Deletion	chr5-156184671 GC > G	8	c.653_653delC	p.Thr219Profs*6	rs1369919728	LGMD	0	0	P	NA	DC	34	P	[35]
32	Insertion	chr5-156344549 G > GT	3	c.62_63insT	p.Tyr23Ilefs*43	rs886043031	LGMD2I	0	0	P	NA	DC	30	LP	[36]
33	Missense	chr5-156759245 T > C	9	c.728T > C	p.Leu242Pro	-	LGMD	0	0	NA	NA	DC	27	LP	[37]
34	Deletion	chr5-156186242delinsAGA	9	c.711delAGA	p.Ala238Glufs*47	-	DCM	0	0	NA	NA	B	23	VUS	[38]
35	Deletion	chr5-156344487AGAT > A	1	c.1_3del	p.Met1?	-	LGMD	0	0	NA	P	B	18	VUS	[39]
36	Splicing	chr5-156647537 G > T	IVS8	c.575 + 1G > T	-	-	LGMD	0	0	NA	VUS	DC	34	LP	[39]
37	Deletion	chr5-156647485 CT > C	7	c.525delT	p.Lys176Asnfs*3	-	LGMD	0	0	NA	NA	DC	32	LP	[40]
38	Deletion	chr5-156508611 GAAAC > G	4	c.204_207del	p.Asn69*	-	DMD	0	0	NA	P	DC	32	LP	[41]
39	Deletion	chr5-156589288 CAGACT > C	5	c.354_358del	p.Thr119Serfs*17	rs1760626889	LGMD	0	0	P	P	DC	30	P	[39]
40	Insertion	chr5-156594970 G > GT	6	c.422dup	p.Thr143Asnfs*13	rs2113389287	LGMD	0	0	LP	P	DC	28	P	[39]
41	Splicing	chr5-156757705 G > T	IVS8	c.699 + 1G > T	-	rs1554137130	DCM	0	0	LP	P	DC	32	LP	[39]

*B* Benign, *LB *Likely Benign, *P* Pathogenic), *NA* Not Available, *VUS* Variant with Uncertain Significance, *LP* Likely Pathogenic, *DC* Disease Causing, *SDC* Sudden Cardiac Death, *IVF* Idiopathic ventricular fibrillation, *TAPVR* Total Anomalous Pulmonary Venous Return, *LGMD* Limb-Girdle Muscular Dystrophy, *DCM* Dilated Cardiomyopathy, *LVNC* Left Ventricular Noncompaction, *IVS* Intervening Sequence

## Discussion

The identification of a novel *SGCD* variant (c.644 A > T, p.Asn215Ile) in a familial DCM case is a significant finding that expands the spectrum of genetic alterations linked to this condition. *SGCD* encodes δ-sarcoglycan, a critical component of the dystrophin-associated glycoprotein complex (DGC) that stabilizes muscle cell membranes. Heterozygous variants in *SGCD* have been documented in a small subset of patients with dilated cardiomyopathy who notably lack skeletal muscle involvement. In the present family, the proband’s phenotype – early-onset dilated cardiomyopathy with no signs of limb-girdle muscular dystrophy – is consistent with these prior observations. The variant was inherited from the proband’s father, underlining a familial pattern of disease transmission. Although the father’s clinical status was not fully described, this inheritance finding suggests the variant may contribute to disease onset and aligns with an autosomal-dominant pattern. The absence of the variant in population databases (e.g., gnomAD, 1000 Genomes) and its novel occurrence further support its potential pathogenic role. Taken together, these findings implicate p.Asn215Ile as a putative disease-causing alteration in the *SGCD* gene and highlight its possible contribution to the proband’s DCM. An additional interpretative challenge in this case is the temporal association between SARS-CoV-2 infection and the recognition of left ventricular dysfunction. Because no pre–COVID-19 echocardiographic data were available, we cannot definitively determine whether the reduced EF during hospitalization represented a de novo, infection-related myocardial injury, or the unmasking of a pre-existing cardiomyopathy. Previous studies have shown that COVID-19 can be accompanied by reduced EF and impaired myocardial function in patients without a known history of heart disease, indicating that viral infection alone may account for part of the observed phenotype in some individuals. In our family, however, the presence of the same *SGCD* variant in the proband and her father, together with the known role of δ-sarcoglycan in cardiomyocyte membrane stability, suggests that COVID-19 most likely acted as a precipitating or aggravating factor in a genetically susceptible myocardium rather than the sole cause of the cardiomyopathic phenotype [9].

An important aspect of our family is the apparent phenotypic discordance between the proband and her father, who carries the same *SGCD* variant but is currently asymptomatic. This pattern is in line with the well-recognized age-dependent and incomplete penetrance of familial DCM, where some genotype-positive individuals may remain clinically silent or develop overt disease only later in life. For heterozygous *SGCD* variants, previous reports have also described variable cardiac expression without skeletal muscle involvement, suggesting that additional genetic and environmental modifiers influence disease onset and severity. In our case, it is plausible that COVID-19 infection acted as a precipitating trigger that unmasked an underlying *SGCD*-related susceptibility in the child, whereas the father has so far not experienced a comparable stressor, underscoring the need for longitudinal follow-up of genotype-positive but phenotype-negative relatives [42].

Previous reports have linked δ-sarcoglycan variants to cardiomyopathy, and the newly identified p.Asn215Ile variant shares both similarities and key differences with known variants. Notably, Tsubata et al. (2000) first reported heterozygous *SGCD* variants in dilated cardiomyopathy, including a missense change (p.Ser151Ala) in an affected family and a 3-basepair deletion removing Lys238 in a sporadic case [42]. Both of those variants reside in the extracellular domain of δ-sarcoglycan (like Asn215Ile) and were shown to disrupt the sarcoglycan complex, as evidenced by reduced δ-sarcoglycan staining in cardiac muscle. Similarly, a novel Arg71Thr (R71T) missense variant in *SGCD* was later identified in a patient with familial DCM, and another DCM-linked variant (Arg97Gln) has been described in the literature. All these cardiomyopathy-associated *SGCD* variants are missense substitutions or small in-frame deletions, mirroring our finding of a single amino acid change. The p.Asn215Ile variant represents a substantial physicochemical alteration – replacing a polar asparagine with a nonpolar isoleucine – akin to the polarity or charge shifts seen in R71T (basic to polar) and S151A (polar to nonpolar) variants. Importantly, like the previously reported δ-sarcoglycan variants, the Asn215Ile change is present in only one allele (heterozygous state) in our proband. This is in contrast to the biallelic *SGCD* variants that cause limb-girdle muscular dystrophy type 2 F (LGMD2F) [43], underscoring a key difference in genetic mechanism between isolated cardiomyopathy and dystrophy phenotypes. Our novel variant has not been reported in prior DCM or muscular dystrophy cohorts, highlighting its uniqueness. Nonetheless, its location in the protein and predicted deleterious nature align closely with established pathogenic *SGCD* variants associated with DCM. This concordance with earlier variants strengthens the case that SGCD p.Asn215Ile may indeed be disease-causing, while its novelty adds new insight into the mutational landscape of *SGCD* in cardiomyopathy.

**Fig. 4:**
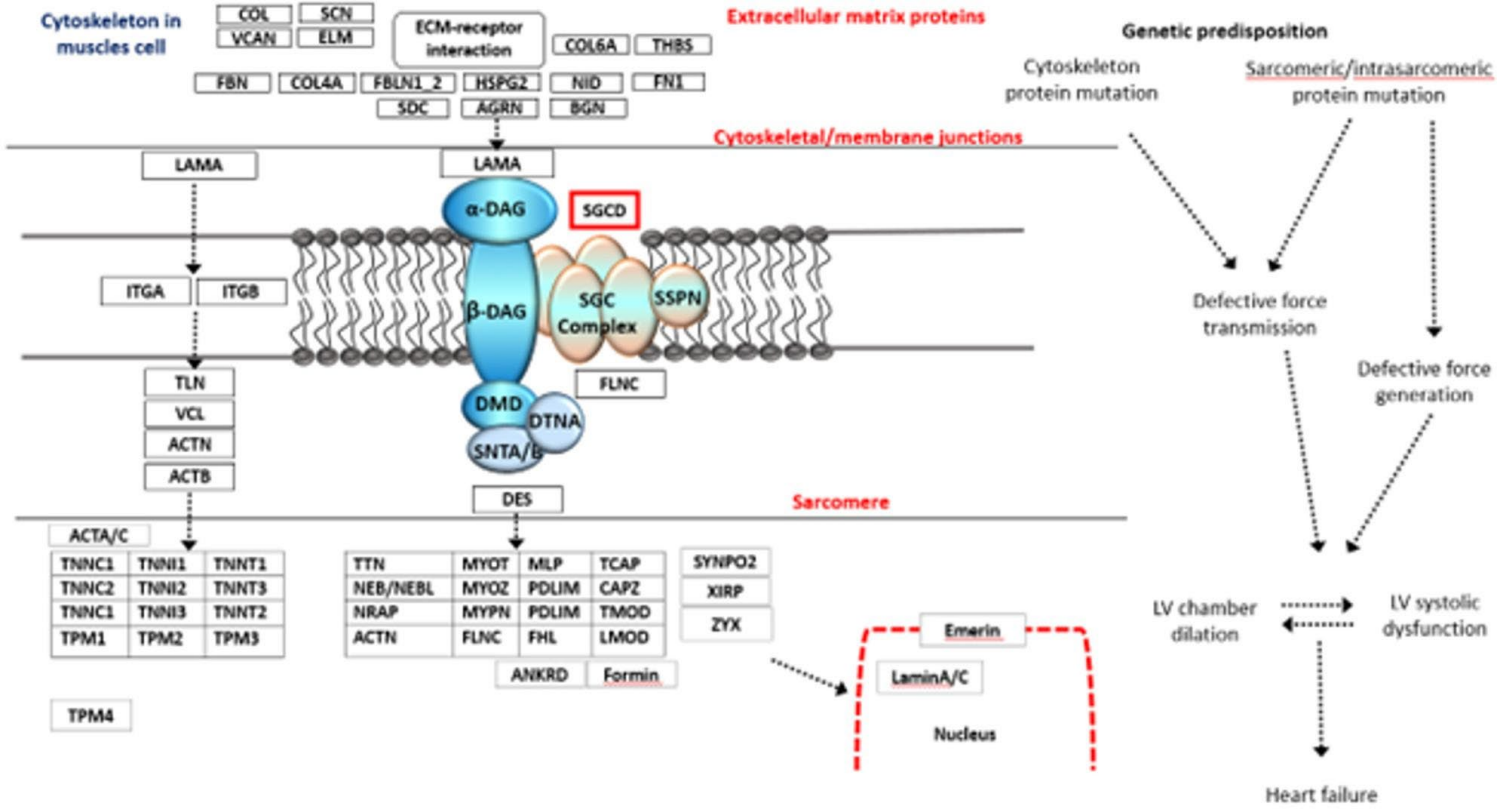
Schematic representation of δ-sarcoglycan within the dystrophin–glycoprotein complex. The figure illustrates the localization of δ-sarcoglycan in the sarcolemma, its interaction with other sarcoglycan subunits and dystrophin, and the approximate position of the p.Asn216Ile variant within the extracellular region, highlighting its potential impact on complex stability and muscle cell membrane integrity

From a pathophysiological standpoint, the p.Asn215Ile variant is predicted to impair δ-sarcoglycan function and thereby compromise cardiac muscle integrity. δ-Sarcoglycan is an asparagine-linked glycosylated single-pass transmembrane protein that, together with other sarcoglycans, forms a subcomplex essential for linking the cytoskeleton to the extracellular matrix via the DGC [44]. Missense variants in this protein can have several deleterious consequences. First, the affected residue (Asn215) is highly conserved across species (as shown by our sequence alignment analysis), suggesting it plays a critical role in the protein’s structure or interaction interfaces Fig. 4. Substitution of asparagine with isoleucine at this position represents a significant change in amino acid properties – from a polar, hydrophilic residue to a hydrophobic one – which may disrupt local folding, eliminate hydrogen bonding, or alter post-translational modification of the protein. Indeed, prior functional studies have shown that DCM-associated δ-sarcoglycan variants can act in a dominant-negative manner on the DGC [45]. For example, cardiomyocytes expressing mutant δ-sarcoglycan (such as R71T or R97Q) exhibit normal sarcolemmal localization of the protein but display reduced membrane stability under mechanical stress. In the case of R71T, the variant introduced an aberrant N-glycosylation site leading to improper glycosylation of δ-sarcoglycan, further impairing its function. By analogy, the Asn215Ile variant could disturb normal glycosylation patterns or protein conformation, potentially hindering the assembly or stability of the sarcoglycan complex. A weakened sarcoglycan–dystrophin connection would render cardiomyocyte membranes more susceptible to damage during contraction-relaxation cycles. This mechanism is consistent with the loss of function effect observed in other δ-sarcoglycan variants and could explain the development of DCM in carriers of the p.Asn215Ile variant. In silico tools lend support to this hypothesis: MutationTaster classified p.Asn215Ile as “disease-causing,” and pathway enrichment analysis (KEGG) identified *SGCD* as significantly involved in dilated cardiomyopathy signaling networks (*p* ≈ 9 × 10^−18), underscoring the gene’s pivotal role in cardiac muscle health. Thus, although direct biochemical assays were not performed in this study, the conserved nature of the Asn215 residue and our understanding of δ-sarcoglycan’s role in the cardiomyocyte membrane suggest that the p.Asn215Ile variant is likely to adversely affect protein function, compromising cardiac muscle fiber integrity and triggering DCM pathogenesis.

The discovery of a novel *SGCD* variant in a familial DCM context carries important clinical implications for diagnosis and patient management [46]. Firstly, it broadens the genetic spectrum of dilated cardiomyopathy, indicating that sarcoglycan gene variants, though rare, should be considered in genetic testing panels for cardiomyopathy. In practice, this means that patients with idiopathic or familial DCM could benefit from expanded genetic screening that includes not only the more common DCM genes (like *TTN*, *LMNA*, etc.) but also genes traditionally associated with muscular dystrophies (e.g., *SGCD*). Early genetic diagnosis can facilitate risk stratification: identification of a potentially pathogenic *SGCD* variant in an index patient prompts cardiological evaluation of relatives even if they have no symptoms. In our case, the father was found to carry the same variant, supporting the need for family screening. All first-degree relatives of a DCM patient carrying an *SGCD* variant should undergo clinical assessment (echocardiography, ECG) and genetic testing if the variant’s significance is strongly suspected. This proactive approach can lead to early detection of cardiac enlargement or dysfunction in presymptomatic carriers, enabling timely intervention. Additionally, the presence of a pathogenic or likely pathogenic *SGCD* variant in a family may warrant tailored follow-up, such as more frequent cardiac monitoring or preventive measures (for instance, exercise moderation or early use of heart-failure medications) to mitigate disease progression. It also raises the consideration of implantable cardioverter-defibrillators (ICDs) if arrhythmic risk is deemed high, as DCM due to cytoskeletal protein defects can sometimes predispose to arrhythmias [13]. Beyond direct patient care, our findings underscore the necessity for genetic counseling. Counseling should inform the family about the autosomal dominant inheritance pattern – each child of an affected parent has a 50% chance of inheriting the variant – and discuss the variable expressivity and incomplete penetrance that might be observed (illustrated by cases where a variant carrier may be asymptomatic). In the scenario that the proband’s father is currently asymptomatic despite carrying the variant, counseling becomes even more crucial to explain the uncertainty: the variant might act as a risk factor that requires additional triggers or age-related factors to manifest disease. Overall, integrating this *SGCD* variant into the family’s clinical management plan exemplifies precision medicine in action, allowing for personalized surveillance and preventative strategies in familial DCM.

While the association of the *SGCD* p.Asn215Ile variant with DCM in this family is compelling, our study has several limitations that must be acknowledged. A primary limitation is the lack of functional assays to directly demonstrate the variant’s impact on δ-sarcoglycan protein function. Our conclusions are based on computational predictions and genotype-phenotype correlation, which, although strong, do not provide the same level of evidence as in vitro or in vivo functional studies. Consequently, according to the American College of Medical Genetics and Genomics (ACMG) criteria, p.Asn215Ile is currently classified as a variant of uncertain significance (VUS) in the absence of definitive pathogenicity evidence. In the absence of dedicated functional assays, our interpretation of the p.Asn215Ile variant in *SGCD* necessarily relies on an integrated clinical–genetic and in silico framework rather than direct experimental validation. Although we were not able to perform cell-based or animal-model functional studies within the current clinical and infrastructural constraints of our center, several converging observations support a likely deleterious effect of this alteration. The variant is extremely rare (absent from major population databases), affects a highly conserved residue in δ-sarcoglycan, is predicted to be disease-causing by multiple bioinformatic tools with a high CADD score, resides in an exon enriched for previously reported cardiomyopathy-associated *SGCD* variants, and segregates with disease in the family. Nonetheless, in line with ACMG recommendations, we conservatively classify p.Asn215Ile as a variant of uncertain significance and clearly acknowledge that future targeted functional studies will be crucial to definitively elucidate its mechanistic impact and to upgrade its classification toward likely pathogenic or pathogenic status, if appropriate.

## Conclusion

In this study, the role and importance of genetic testing alongside clinical diagnosis were identified in improving the diagnosis of the disease in affected individuals, recognizing carriers within the family members, and enabling prenatal prevention.

## Data Availability

No datasets were generated or analysed during the current study.
